# Do Italian Companies Manage Work-Related Stress Effectively? A Process Evaluation in Implementing the INAIL Methodology

**DOI:** 10.1155/2015/197156

**Published:** 2015-10-04

**Authors:** Cristina Di Tecco, Matteo Ronchetti, Monica Ghelli, Simone Russo, Benedetta Persechino, Sergio Iavicoli

**Affiliations:** Department of Occupational and Environmental Medicine, Epidemiology and Hygiene, Italian Workers' Compensation Authority (INAIL) Research Area, Via Fontana Candida 1, Monte Porzio Catone, 00040 Rome, Italy

## Abstract

Studies on Intervention Process Evaluation are attracting growing attention in the literature on interventions linked to stress and the wellbeing of workers. There is evidence that some elements relating to the process and content of an intervention may have a decisive role in implementing it by facilitating or hindering the effectiveness of the results. This study aimed to provide a process evaluation on interventions to assess and manage risks related to work-related stress using a methodological path offered by INAIL. The final sample is composed of 124 companies participating to an interview on aspects relating to each phase of the INAIL methodological path put in place to implement the intervention. INAIL methodology has been defined as useful in the process of assessing and managing the risks related to work-related stress. Some factors related to the process (e.g., implementation of a preliminary phase, workers' involvement, and use of external consultants) showed a role in significant differences that emerged in the levels of risk, particularly in relation to findings from the preliminary assessment. Main findings provide information on the key aspects of process and content that are useful in implementing an intervention for assessing and managing risks related to work-related stress.

## 1. Introduction

Psychosocial risks are widely recognised as emerging risks to the health and safety of workers and are linked to workplace problems such as work-related stress, harassment or bullying, and workplace violence [1]. They are one of the most challenging issues to be faced, not only because of their widespread increase in Europe, but also in consideration of the significant related socioeconomic costs not only for companies but for society as a whole [2].

The latest pan-European opinion poll on Occupational Safety and Health (OSH) conducted by the European Agency for Safety and Health at Work [3] reported that 51% of workers consider work-related stress common in their workplace. Furthermore, four out of ten workers sustained that stress was not managed adequately in their organisation. A recent survey on health and safety at work carried out by INAIL on over 8000 Italian workers indicated that workers generally feel more exposed to work-related stress than to any other risk in the workplace [4]. Since the 1970s, studies have been developed to investigate psychosocial risks and their impacts and to provide practical solutions at the organisational and policy levels to manage them [5].

On the European level, efforts have been made to guide companies in assessing and handling these risks, including techniques for developing strategies and tools for managing them. The European Framework Agreement (2004) provides employers and employees with a reference framework for identifying, preventing, and managing work-related stress on the organisational level. This has since been accompanied by a series of methodological proposals from different European countries to offer companies solutions that are both effective and sustainable in managing psychosocial risks at work.

In Italy, the inclusion in the specific OSH legislation (Legislative Decree 81/08 and amendments) of the World Health Organization definition of health as a “state of complete physical, mental, and social wellbeing and not merely the absence of disease or infirmity” [6] served as the basis for protection against psychosocial risks at work, particularly those related to work-related stress [7]. This Legislative Decree, implementing the European Agreement on work-related stress, established the employer's responsibility for assessing and managing risks related to work-related stress, with the collaboration of company OSH professionals. This has led to the need for scientific strategies and effective tools to enable the company to fulfill these requirements using sustainable risk management strategies, in compliance with the guidelines provided by the Permanent Consultative Commission for Occupational Health and Safety [8].

Drawing on its national and international research on the issue, in 2011, the INAIL Research Area developed a methodological proposal for assessing and managing risks related to work-related stress [9]. The proposal reflects the minimum requirements and the methodological criteria identified in regulatory terms [10] and it is based on the risk management framework. It therefore starts by identifying and estimating/measuring the risk and identifies what should be considered the key resources, strategies, and measures for correcting, controlling, and preventing it, using a participatory process [10–12]. It offers a dynamic path, made up of four key phases and based on a continuous improvement cycle [13], which should involve company OSH professionals and the active participation of workers right from the initial planning stages.

Offering tools that are scientifically proven and easy to use [8, 14], the INAIL approach has now been employed by a large number of Italian companies (more than 6000) in the public and private sectors, in many different fields of business (health, services, education, construction, etc.).

## 2. The Four Phases of the Methodological Path

The INAIL methodology represents an intervention for assessing and managing the risks related to work-related stress in four phases, each with its own specific objectives, activities, and supporting tools (Table 1). The first, preliminary phase, outlines activities for planning and managing the entire process of risk assessment, which is essential for ensuring the accuracy and effectiveness of the subsequent phases. In addition to establishing a steering group responsible for planning the assessment process, during this phase, it is important to ensure workers are involved, using not only communication strategies but also training, if necessary, for those concerned. Lastly, during this phase, homogeneous groups of workers are identified (see [8] for a full definition of homogeneous groups) on which to implement the methodological path.

The second phase is the actual preliminary assessment, analysing the outcome indicators, consisting of sentinel events, work content factors, and work context factors linked to the work-related stress [15]; these objective and/or verifiable indicators are gathered using a checklist compiled by each homogeneous worker group. The third phase, in-depth assessment, comprises a detailed analysis of work content and context factors, from the workers' point of view. The Italian version of the HSE (Health and Safety Executive) Indicator Tool is available for this phase [16], but additional tools or ones that may be more suitable (focus groups, semistructured interviews, and meetings) can also be used depending on the characteristics of the company making the assessment (e.g., small enterprises, specific economic sectors).

The fourth and final phase involves managing the conditions of risk that have come to light in the previous phases, developing corrective or preventive actions, and verifying their effectiveness, based on the outcomes of risk assessment. The final phase also aims to develop a risk monitoring plan, which will allow for a new cycle of intervention two or three years from the conclusion of the previous one, as required by the cyclical, dynamic nature of the path offered [11, 13].

## 3. This Study

Despite the fast-growing numbers of methodological approaches to assess and manage this kind of risk and to confirm the effectiveness of their outcomes in methodological terms [8, 13], only very few studies have fully analysed the process implemented by companies to assess and manage risks related to work-related stress, aimed at understanding how the intervention has been implemented and its main impacts on the effectiveness of the results [16].

Some UK studies in this direction have explored progress in implementing the Management Standards approach, developed and offered to companies by the Health and Safety Executive [17, 18]. As defined by Mellor and colleagues (2011) “the detailed processes through which a programme has unfolded can explain its success or failure” (page 1041). It follows that investigating the ways of implementing an integrated approach for assessing and managing risks related to work-related stress can be useful for verifying how appropriate the actual process has been, also in view of the ultimate goal of adequately detecting the risk being investigated and avoiding false conclusions and inconsistent results [16, 19]. So detailed analysis is needed to identify those elements of the process that are needed to ensure its effectiveness and, if lacking, put its validity at risk.

This analysis refers to studies on Intervention Process Evaluation, a topic that is attracting growing attention in the literature on interventions linked to stress and the wellbeing of workers [16, 20–22]. Previous studies indicate some key factors related to the process and content of an intervention to manage stress in the workplace, making for its effective implementation. These factors are significant for assessing the actual measures taken but also for assessing the processes employed to put the measures into place [19, 22]. Some also refer to guiding principles and typical aspects of the risk management approach that should be considered when implementing methods based on these strategies [13, 23]. A brief overview of the main ones follows.

Any intervention stands or falls on the ability to plan and conduct the process from the perspective of project management [18, 24]. As mentioned previously, the INAIL methodology requires the establishment of an assessment management group, or steering group, responsible for planning and managing the assessment, and including a nominated manager as well as OSH professionals, including workers' representatives for safety. This group has key functions in correctly implementing the process (Table 1), many of which are linked to the success of the intervention and have also been defined as crucial in the guidelines drawn up by national supervisory bodies [25].

The success of an intervention depends not only on how the process is managed upstream, but also on management support during the intervention (e.g., information and clear communication with staff) and the active participation of managers [18, 24]. Several studies have also indicated that the active involvement of workers [21, 26] plays a key role in the success of interventions. In any methodological path for assessing and managing work-related stress, workers' participation contributes to the correct estimation of risk, as they are an essential source of information about their own working conditions. Their involvement also aims to boost the levels of knowledge and internal skills with a view of creating a cycle of continuous improvement [10].

Another key factor is the level of specific skills in assessing the risk and stress of the people managing the process [27]. If these are inadequate, specific training must be laid on for those involved.

In some cases, companies may use external consultants when implementing an intervention [18]. However, one basic criterion for developing the INAIL method is that companies should be able to implement the assessment and management process autonomously. Naturally, however, companies can decide to use an external consultant if they deem it essential.

Another enabling factor in the approach to assess and manage the risks related to work-related stress is the possibility of combining tailored and contextualized tools with standard ones to assess the needs linked to the specifics of each organisation [18]. The INAIL methodology was developed with modularity and flexibility in mind, allowing for the use of supporting tools to achieve comprehensive risk assessment in compliance with the each company's specific features (e.g., size, business sector).

In line with evidence from previous studies mentioned above, the aim of this study was to assess the implementation of the INAIL methodology in a large sample of Italian companies. Thus, first of all, we explored the presence of factors reported in the literature as related to the process evaluation of an intervention to manage stress in the workplace (namely, the company's ability to plan and manage the process, training, OSH professionals and workers' involvement, possibility of combining tailored and contextualized tools, etc.). Then, we analysed the relationship between these factors and the findings from the two assessment phases to understand their impact on changes in risk levels. Finally, we explored the perceptions of the usefulness of the whole methodological path and its assessment phases.

Studies that verified the validity and soundness of the supporting tools [8, 14, 28] led to this follow-up analysis of the processes put into place by companies for implementing the methodological path proposed by INAIL.

## 4. Materials and Methods

The sample of companies involved in this study was extracted from the INAIL web platform database [8]. Two main criteria were followed in selecting them: (1) companies that had already completed the INAIL methodological path and had therefore used both the checklist and the Indicator Tool for the assessment phases; (2) companies where the homogeneous groups comprised more than six workers, for methodological reasons relating to the use of the Indicator Tool.

The resulting sample consisted of 339 companies that had employed a work-related stress assessment and management intervention using INAIL methodology. We sent these companies a letter describing the investigation and its purpose, contents, how it would be conducted, and how data would be handled. The letter asked them if they would complete a questionnaire presented during a telephone interview with an occupational psychologist. Of the 339 companies, 124 agreed to the interviews (37% response rate); this gave 330 homogeneous groups of workers, meaning 330 checklists and 4500 questionnaires.

Most of the companies had up to 50 employees (22% from 1–9 and 40% from 10–50), 22% from 51–250 and 16% more than 251. The five most frequent business sectors were services (21.8%), manufacturing (17.7%), professional sector, healthcare and social welfare (16.9%), scientific and technical ones (12.1%), and construction (5.6%).

INAIL occupational psychologists conducted the phone interviews with an internal representative of the companies in the work-related stress assessment steering group. The questionnaire comprised 22 items to analyse aspects relating to each phase of the method. Some of the questions investigated qualitative aspects of the assessment and related to the perceived level of usefulness of these aspects (using a Likert type scale from 1 = completely useless to 5 = completely useful) and of the method as a whole and any difficulties that had been met. Other questions were designed to investigate how the single phases of the assessment process were carried out and are in fact the most significant items for achieving the objectives of the follow-up analysis.  Table 2 shows some examples relating to the various aspects.

In addition to the data collected during the interviews, the results of the two assessment phases were extracted from the web platform; these were the checklist findings and the Indicator Tool, for each homogeneous group in the companies involved. They are described below.

### 4.1. Preliminary Assessment Results

A checklist was compiled for each homogeneous group assessed [8] to gather a range of indicators (sentinel events, work content, and context factors) on a dichotomous scale. Each statement in the checklist contributes to an overall score. The sum of the scores for the three areas establishes the position of the homogeneous group based on a table of levels of risk: low, medium, and high.

### 4.2. In-Depth Assessment Results

The self-report questionnaire used to obtain details is the Italian version of the UK HSE Management Standards Indicator Tool [14, 28]. It comprises 35 items measuring the seven dimensions (Management Standards) used for describing the indicators of work context and content, corresponding to seven ideal conditions/states to be achieved for the prevention and reduction of the risks related to work-related stress in companies. The output is a profile of the levels of risk of each homogeneous group for each of the seven dimensions of the questionnaire.

## 5. Analysis

Data were analysed using the IBM SPSS Statistics version 21. Percentage frequencies were calculated for the multiple-choice questions based on the total number of answers, and comparisons with other questions in the follow-up questionnaire were made by processing the double-entry tables. For questions without multiple-choice responses, parametric tests such as the *t*-test and ANOVA were used to verify relations between the variables compared. Nonparametric tests were also used such as Chi-square (*χ* ^2^) and the Kruskal-Wallis test, a one-way analysis of variance by ranks which is considered the nonparametric equivalent to the ANOVA and establishes whether the difference(s) between the medians for one or more subsamples are due to chance or are statistically significant. The samples and the subsamples were verified for normality of distribution using the *p*-Shapiro-Wilk test, so as to establish how to consider the parametric and nonparametric statistics each time. We took *p* < 0.05 as significant.

## 6. Results

In keeping with the aims of the study, the results for some key factors that have emerged from the literature as related to successfully assessing and managing risks related to work-related stress are presented below, including the relationship between such factors and findings from the two assessment phases. For easier reading, they are set out following the phases of the INAIL methodological path (Table 2).

### 6.1. The Preliminary Phase

Of the 124 companies interviewed, 97.4% (115) confirmed they had completed the preliminary phase. Although only nine stated they had not completed it (2.6%), we compared the levels of risk obtained in the preliminary assessment phase with those of the responders that had completed the preliminary phase, to check for significant differences (Table 3). All the tests indicated a significant difference between the two groups; in particular, the preliminary assessment appeared more positive, that is, tending towards low risk, in the companies that had not completed the preliminary phase.

Table 4 shows the main indicators investigated in the interviews in the preliminary phase of the INAIL methodological path. There was a high level of workers' participation. The companies chose to involve a representative sample of workers in 32.2% or all workers in 39.3%; in 27.4%, only the workers' representative for safety was involved. Nearly three quarters of the companies (74%) interviewed provided specific training for those involved in interventions. The majority of respondents considered this extremely useful in developing the risk assessment and management process, and only 3.2% rated it as of little or no use.

### 6.2. Preliminary Assessment Phase

As part of the preliminary assessment, we investigated indicators of the involvement of OSH professionals in the planning phase and in completing the checklist and checked for any difficulties encountered in completing the checklist (Table 5). Personnel involved included those responsible for health and safety management and then the employer, in keeping with the approach taken when assessing other risks in the company. Workers (60% of respondents) and/or their safety representative (68%) were frequently involved. In particular, workers were involved in the briefing for communicating the measures taken by the company, but also gathering, analysing, and discussing the data from the checklist.

Just over a third of companies (35%) stated they did not involve their workers or workers' safety representatives; 22% engaged an external consultant for implementing this phase. In keeping with the objectives of the study, we made detailed analyses to check for significant differences in the findings of the preliminary assessment phase linked to the participation of workers or their safety representatives, and to the involvement of an external consultant in the process (Table 6).

There was a significant tendency towards higher levels of risk in companies that involved workers and/or their representatives. In contrast, assessments with lower levels of risk tended to come from companies that engaged an external consultant for implementing the phase.

Although the majority of companies stated they had no difficulty in completing the checklist (57%), those that did encounter some problem referred in particular to its applicability to their business context for all three families of stress indicators.

### 6.3. In-Depth Assessment Phase

Among the reasons to implement the in-depth assessment phase, around 43% of the companies wanted to analyze workers' perceptions of risks related to work-related stress, 33.8 % wanted to obtain details of the preliminary assessment findings to define risk more clearly, and 20.5% wanted to better identify the corrective measures to be put in place. Only 1.5% of companies implemented this phase as a result of the ineffectiveness of corrective measures taken following the preliminary assessment, a process required in order to comply with regulatory requirements. However, detailed analysis did not bring to light any direct link between the results of the preliminary assessment and the reasons that prompted companies to implement in-depth assessment.

In 56%, the use of further tools in addition to the Indicator Tool offered in the INAIL method was confirmed. In 25.3%, focus groups were formed for samples of employees, in 18.7% for detailed meetings and in 12.0% for semistructured interviews.

Lastly, the interviews showed a high level of appreciation (M 3.48, SD 0.854) regarding the comprehensiveness of the results from the in-depth phase for clearly defining the risks related to work-related stress.

### 6.4. Corrective Measures and Monitoring

Developing corrective measures and actions is a key step in this methodological process. The conditions of risk that emerge from the previous phases must be managed by defining and implementing corrective or preventive measures and verifying their effectiveness, on the basis of risk assessment. The majority of respondents had adopted or were currently adopting (51.6% and 20.7%, resp.) corrective action or measures to prevent, reduce, or eliminate conditions of psychosocial risks.  Table 7 illustrates the action taken by the respondent companies, based on the types classified in the European Framework Agreement on work-related stress (2004).

As regards the companies' perceptions of the usefulness of the INAIL methodology, most of them reported they found it useful in assessing and managing the risks related to work-related stress (M 3.62, SD 0.771). Companies also reported positive perceptions of the usefulness for the preliminary phase (M 3.57, SD 0.785) and the use of* ad hoc* tools for identifying the homogeneous groups (M 3.18, SD 0.724). Lastly, the interviews indicated a high level of appreciation of the exhaustiveness of the results gathered in the in-depth phase for clearly defining the risks related to work-related stress (M 3.48, SD 0.854).

## 7. Discussion

This study makes a process evaluation of interventions for the assessment and management of risks related to work-related stress, using a methodological path offered by INAIL involving the investigation of (1) factors that contribute to its effective implementation, (2) the impact of these factors on changes in the level of risk, and (3) perceptions of the usefulness of the methodological path and the assessment phases.

In the follow-up interviews on a sample of companies that followed the entire methodological path, several recurring factors help explain the differences arising during the assessment phases.

In terms of the process, significant differences emerged in the levels of risk resulting from the preliminary assessment phase in companies that completed the preliminary phase, which was the majority of the sample. There was a tendency towards higher levels of risk compared to those that did not complete this phase. Although it is clear that this result must be interpreted with caution, given the small number of companies not completing the preliminary phase, the ability of a company to plan and manage the process from the perspective of project management is recognized as one of the features that is often associated with the success of interventions, in the context of work-related stress and wellbeing in the workplace [24]. The methodological path offered strongly recommends setting up a steering group for planning and managing the preparatory work for actual implementation of the intervention as well as for the involvement of the OSH professionals.

It can be assumed that the differences in the tendency towards risk depend partly on how accurately the assessment is conducted from the initial stages. Crucial steps in the process, such as identifying homogeneous groups of workers, verifying internal skills, the involvement and participation of workers, and their safety representatives, are completed during the preliminary phase. Therefore, thorough preparation of the organisation for risk assessment and a participatory approach probably make it easier to make the best use of the tools and fully recognise any issues related to risk factors.

Internal skills and expertise are another primary aspect of the process, acknowledged in the literature [27]. The majority of companies that completed the preliminary phase considered it necessary to provide specific training on this issue, probably to compensate for a lack of internal expertise. In most cases, this was perceived as extremely useful for completing the process of risk assessment and management. In some cases, this lack was compensated by engaging an external consultant, although the INAIL methodology was developed with a view to ensuring that companies could use it autonomously. There appeared to be a tendency towards assessments with lower risk during the preliminary assessment phase in the companies that engaged external consultants. Therefore, in future, it would be interesting to analyse the impact of external professionals on the assessment process and its outcomes; for example, the types of professionals involved (psychologists, physicians, employment consultants, etc.) and the support methods offered in the steering group could be investigated.

As noted in previous studies [21, 26] and in the indications of the Permanent Consultative Commission regarding the minimum legal requirements for assessing and managing the risks related to work-related stress, the direct participation of workers and/or their representatives is considered decisive in implementing this type of intervention. The companies interviewed attributed substantial importance to the participation of employees and generally included as many as possible right from the early phases of the process, using a strategy of communication and updating. The study findings confirm its importance, indicating a tendency to lower levels of risk in cases where this participation was lacking, particularly in essential steps such as completing the checklist. In this case, it can also be assumed that the greater the participation of workers in the preliminary assessment phase, the more accurate the information on working conditions. In any case, the majority of companies appear to recognise the role of employees in assessing this risk, as they are a valuable source of information about work context and work content.

The findings of the in-depth assessment confirmed this, regardless of the results from the previous phase, with the main aim of analysing workers' perceptions related to organisational risk factors. Most of companies reported low risks related to work-related stress in the preliminary assessment findings. This already indicates a willingness to establish the presence of risk in the most comprehensive manner possible in order to plan any necessary targeted and preventive measures. In this regard, the majority of companies had adopted or were adopting corrective measures to prevent or reduce work-related stress risks, as well as a monitoring plan.

The tools offered appear to provide full information and indications for identifying the different types of intervention, in compliance with the requirements of the European Framework Agreement. However, a limiting factor in this study is the lack of data, in this first phase, on the actual methods used to manage risk, as well as verification of the effectiveness of corrective measures because of the impossibility of following the process step by step in an observational way. This lack of data also reflects a scarcity of specific information about the context in which each company developed the intervention for assessing and managing work-related stress risks, according to the literature on process intervention evaluation [29, 30]. To address this, case studies are now under way in specific contexts (e.g., social and health care, public administration, and small and medium enterprises) designed to analyse in detail the applicability of the methodological path, using the support provided to the steering group throughout all the steps.

The main issue related to content concerns the applicability of the checklist to different business contexts. The proposed checklist was developed as a tool that could be used across a range of different contexts to identify risk factors. Ongoing studies have, in actual fact, brought to light significant differences between the levels of risk emerging from the use of the checklist and the Indicator Tool [31]. During the in-depth assessment phase, additional tools can still be used alongside the Indicator Tool, such as focus groups, which are useful for obtaining more detailed information, as the results show. The majority of the sample considered the tools offered during this phase exhaustive enough to clearly define risk. This confirms the need for a bottom-up type of methodological approach [18] where workers must be involved to successfully identify risk and the relative measures for improvement.

To further develop the methodology, we are now running studies on the contextualisation and adaptability to specific occupational features and needs. As part of a project funded by the Ministry of Health, additional tools will be developed, specifically tailored on the basis of companies' characteristics to take account of their sector and size.

## Figures and Tables

**Table 1:** The four phases of the methodological path.

Phase	Aim	Activities and tools
Preliminary phase	To prepare the organisation for subsequent assessment processes	(i) Establishment of a steering group to manage assessment (employer, managers, OSH professionals, workers, and other health-related organizational figures such as the human resources manager, internal occupational psychologists), (ii) development of a communications/employee engagement strategy (meetings, training, etc.), (iii) drafting the risk assessment plan (scheduling each phase, actions, and players involved), (iv) identification of homogeneous groups of workers on which the assessment is to be made.

Preliminary assessment	To assess objective and verifiable indicators associated with work-related stress under three main headings: (1) sentinel events (e.g., injury rates, absence due to sickness, and turnover), (2) work content factors (e.g., work load, working hours, and working environment), (3) work context factors (e.g., interpersonal relationships work/home interface)	A checklist is compiled for each homogeneous group of workers, with their participation.

In-depth assessment	To assess employees' perceptions about work content/context factors	*Italian version of the Management Standards Indicator Tool* made up of 35 items corresponding to the seven Management Standards: Demands, Control, Managerial Support, Peer Support, Relationships, Role, and Change.

Interventions and monitoring	To manage work-related stress by identifying corrective measures and interventions based initially on the findings from the preliminary assessment. To outline a monitoring plan	*A focus group guide* to help organisations set up focus groups to collect detailed information for interpreting the results of the previous steps and identifying the best solutions.

**Table 2:** Main aspects investigated in implementing the process for assessing and managing risks related to work-related stress.

Preliminary phase	Information and communication strategy Participation/involvement of workers Company and external figures involved Specific training
Preliminary assessment	Company and external figures involved and ways of involvement Specific training Participation/involvement of workers

In-depth assessment	Reasons that led to this phase being conducted Use of additional tools

Interventions and monitoring	Adoption of interventions/corrective measures and types Assessment of the effectiveness of management measures Monitoring plan

**Table 3:** Significant differences in scores from the preliminary assessment for responders that had implemented the preliminary phase.

	M	SD	*p* Shapiro	*t*-test	Kruskal-Wallis	*χ* ^2^
Yes	1.32	0.506	0.000	*F* = 34.370 (0.000)	0.049	3.92 (0.140)
No	1.00	0.000	0.380			

**Table 4:** Descriptive indicators related to the preliminary phase of the INAIL's methodology to assess and manage the risks related to work-related stress (number of companies interviewed = 124).

Preliminary phase	
Implemented	
Yes	97.4%
No	2.6%
	100.0%
Perceived usefulness	
Mean (ranking 1–5)	3.57
Standard deviation	0.785

Involvement	

Workers' involvement	
Yes	85.7%
No	14.3%
	100.0%
Target of the strategy	
Workers' representatives for safety	27.4%
Trade union representatives	1.0%
All workers	39.3%
A sample of workers	32.3%
	100.0%
Way of involvement	
Meetings	65.3%
E-mail	7.8%
Intranet alerts	5.4%
Posts on the bulletin board	5.8%
Brochure	15.7%
	100.0%

Training	

Specific training	
Yes	73.8%
No	26.2%
	100%
Type of training	
Traditional course	85.9%
E-learning	14.1%
	100.0%
Perceived usefulness of training	
Mean (ranking 1–5)	3.50
Standard deviation	0.741

Identification of homogeneous	
groups of workers	

Use of *ad hoc* tools	
Yes	74.4%
No	25.6%
	100.0%
Perceived usefulness of *ad hoc* tools	
Mean (ranking 1–5)	3.18
Standard deviation	0.724

**Table 5:** Descriptive indicators related to the preliminary assessment phase of the INAIL's methodology to assess and manage the risks related to work-related stress (number of companies interviewed = 124).

Preliminary assessment phase	
Involvement	

Personnel involved	
Employer	14.6%
Manager as employer's delegate	8.2%
Personnel assigned	7.8%
Health and safety manager	18.9%
Workers' safety representatives	15.7%
Health and safety workers assigned	4.3%
Company physician	11.6%
Workers	13.8%
External consultant	5.0%
Total	100.0%

Workers' involvement	
In the information meetings	34.1%
In planning the assessment	15.2%
In the collection, analysis, and discussion of data from the checklist	33.8%
In identifying corrective measures	16.9%
Personnel completing the checklist	
Employer	15.2%
Manager as employer's delegate	11.1%
Health and safety manager	23.8%
Company physician	10.7%
Health and safety workers assigned	5.3%
Workers' safety representatives	18.1%
Workers	15.8%
Total	100.0%
Problems in completing the checklist	
Yes	42.6%
No	57.4%
	100.0%

Concerns that emerged	

Type of concern in completing the checklist	
*Sentinel event*	37.7%
Content of statements not clear	2.8%
Concerns about the application to different business contexts	37.3%
Concerns about data availability	22.2%
	100.0%
*Work content factors*	38.6%
Content of statements not clear	12.0%
Concerns about the application to different business contexts	36.9%
Concerns about data availability	12.4%
	100.0%
*Work context factors*	36.1%
Content of statements not clear	10.2%
Concerns about the application to different business contexts	40.3%
Concerns about data availability	13.4%
	100.0%

**Table 6:** Comparison of the preliminary assessment for companies that involved workers or their safety representative and for those that engaged external consultants.

	M	SD	*χ* ^2^	Shapiro-Wilk *p*	ANOVA	*t*-est	Kruskal-Wallis
Workers involved							
Yes	1.34	0.513	3.941 (0.139)	0.000	0.047	0.014	0.05
No	1.16	0.374		0.000			

Consultants engaged							
Yes	1.22	0.450	4.101 (0.129)	0.000	0.053	0.038	0.04
No	1.35	0.517		0.000			

**Table 7:** Descriptive indicators relating to the corrective measures and monitoring phase of the INAIL's methodology to assess and manage the risks related to work-related stress (number of companies interviewed = 124).

Corrective measures and monitoring	
Implementation of interventions	
Yes	51.6%
No	20.7%
In implementation	20.7%
After which phase the interventions were implemented	
Preliminary assessment	9.8%
In-depth assessment	20.7%
Both of these	69.5%
	100.0%
Type of measure	
Organizational	20.3%
Communication	19.3%
Training	19.5%
Procedural	24.5%
Technical	16.4%
Time from the last assessment	
From 1 to 6 months	19.3%
From 6 to 12 months	42.9%
Over 12 months	37.9%
Monitoring plan	
Yes	61.4%
No	38.6%
Method for implementing the monitoring plan	
Periodic monitoring of sentinel events	21.9%
Periodic monitoring of workers' perceptions	41.8%
Other ways for assessing the effectiveness of the measures	36.3%
